# The Effect of Gonadotropin on Glucose Transport and Apoptosis in Rat Ovary

**DOI:** 10.1371/journal.pone.0042406

**Published:** 2012-08-01

**Authors:** Cheng Zhang, Wanbao Niu, Zhengpin Wang, Xiaoxia Wang, Guoliang Xia

**Affiliations:** 1 College of Life Science, Capital Normal University, Beijing, People's Republic of China; 2 State Key Laboratory for Agro-Biotechnology, College of Biological Science, China Agricultural University, Beijing, People's Republic of China; Institute of Zoology, Chinese Academy of Sciences, China

## Abstract

Although the effects of Gonadotropin on ovarian physiology have been known for many decades, its action on glucose uptake in the rat ovary remained poorly understood. Evidence also suggests that glucose uptake is mediated by a number of glucose transporter proteins (Glut). Therefore, we examined the rat ovary for the presence of Glut1–4 and blood glucose level after eCG (equine chorionic gonadotropin) and anti-eCG antiserum treatment. All of the glucose transports were present in the ovarian oocyte, granulosa cells and theca cells in different stage follicles. The expression of Glut in ovary was up-regulated by eCG, however, anti-eCG antiserum reversed eCG action. Western blot analysis also demonstrated the content of Glut1 was higher in eCG treatment group compared with anti-eCG antiserum and control group. The same tendency was shown in other glut isoforms. Moreover, there were no significant difference between the anti-eCG antiserum and control group. In additional, the level of serum glucose in eCG treatment group was significantly higher than others, which is similar with glut expression pattern. High glucose level in blood is correlated with increased expression of glucose transporter proteins in rat ovary. Meanwhile, anti-eCG antiserum increased granulosa cell apoptosis in antral follicle compared with those in eCG group. Our observations provide potential explanation for the effects of Glut on follicular development in rat ovary and a role for eCG in the regulation of ovarian glucose uptake.

## Introduction

Glucose is an essential metabolic substrate of all mammalian cells for energy demand [1]. It is a hydrophilic molecule, can not permeate the plasma membrane, and its uptake is mediated by a number of glucose transporter proteins (Glut). The main glucose transporter isoforms have been identified, which include thirteen members (Glut1 to Glut12 and HMIT; gene name SLC2A) [2], [3]. These glut isoforms exhibit different substrate specificities, kinetic properties and tissue expression profiles. There are some studies show that Glut1, Glut2, Glut3 and Glut4 have a relatively high affinity for glucose and are the major contributors to glucose uptake, so they are thought to play an important role in tissues highly dependent on glucose as their energy source [4], [5], [6], [7], [8], [9], [10], [11], [12].

The ovary is one of the most dynamic endocrine organs, and follicular development can be classified into three phases according to their development stage and gonadotropin dependence: gonadotropin-independent phase, gonadotropin-responsive phase and gonadotropin-dependent phase [13], [14]. The whole process includes follicle recruitment, selection, and ovulation. In mammals, only 1% primordial follicles are selected to ovulate during ovarian cycle in whole life. A substantial energy source is necessary to sustain its activity. In the rat ovary, the predominant energy substrate appears to be glucose [9], [15].

Importantly, several recent reports have shown that Glut1, 3 and 4 are expressed in the ovary of sheep [16], bovine [6], rat [15], [17] and mouse [18] with considerable species differences in the expression profiles. The reports also demonstrated that the expression of these Gluts are also regulated by intraovarian factors during follicular development, maturation and ovulation, such as E_2_ (Estradiol) [19], [20], IGF (insulin growth factor)-I [18] and interleukin-1 [17] as well as FSH (follicle stimulating hormone) [15]. These results indicate that the ovary requires a regulatory mechanism to regulate its glucose uptake.

However, little is still known the expression pattern of Glut1–4 in rat ovary and whether gonadotropin regulates gluts expression in vivo, which is also related with blood glucose level. In the present study, we have examined different Glut isoforms in rat ovary and demonstrated for the first time that gonadotropin respectively up-regulate Gluts expression, which is correlated with blood glucose level. These gonadotropin responses were appeared to be attenuated by anti-eCG antiserum. TUNEL was used to detected apoptosis cell in rat ovary. The results showed that anti-eCG increased TUNEL positive cells in granulosa cell, not in theca cell, in all type follicles.

## Results

### Gene expression of Gluts in ovary

**Figure 1:**
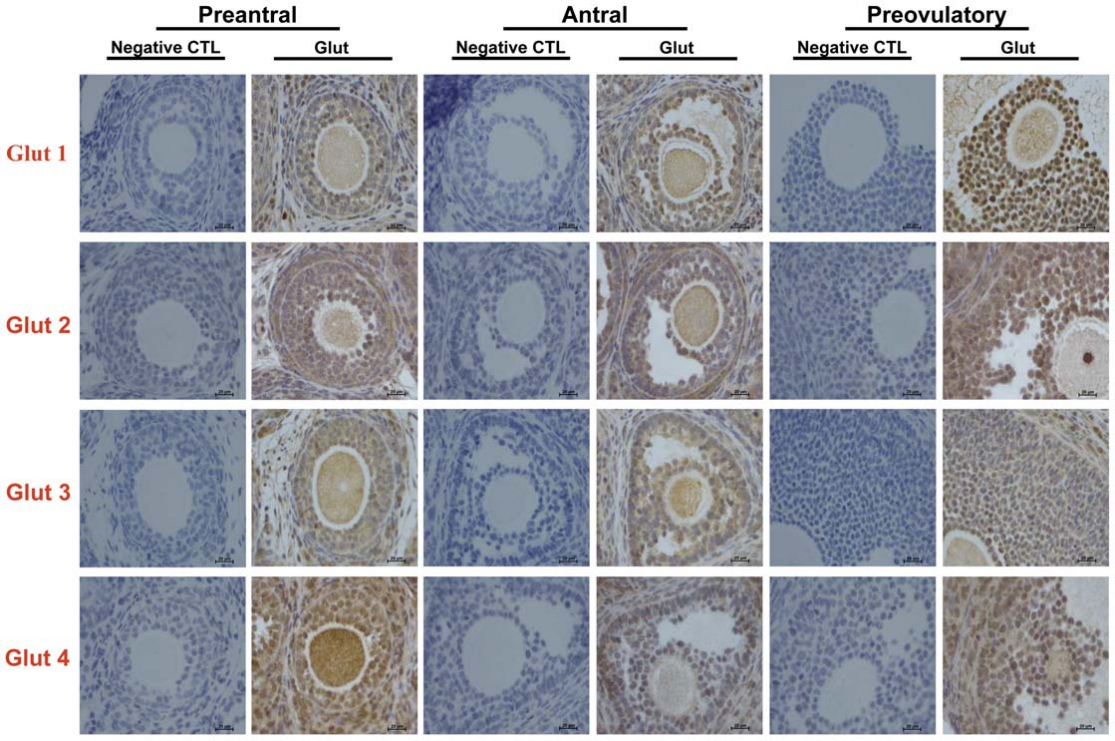
Immunolocalization of Glut1–4 in preantral and small antral follicle in rat. Ovaries from immature rats were fixed and adjacent paraffin sections were cut. Glut1, Glut2, Glut3 and Glut4 proteins were localized with corresponding specific antibodies by ABC (avidin -biotin complex) method. Column Glut show positivities and immunoreactivities for Glut1, Glut2, Glut3 and Glut4 respectively by IHC. Note that Glut1–4 were not only detected in granulosa cells, theca cells but also in oocyte. Each independent data came form six ovaries. Magnification, 400×, Bar = 20 µm.

In order to examine the pattern of different Glut isoforms expressed in rat ovary, IHC was used to detect the Gluts expression in different stage follicle. In the control group, Glut1, Glut3 and Glut4 were not only detected in the theca cells and granulosa cells but also in the oocyte. The results were consistent with the finding that Glut1, Glut3 and Glut4 were detected in rat ovary [17]. However, the expression of Glut isoforms were not in follicular stage manner. Interestingly, as shown in Figure 1, all the cell type displayed positive staining for Glut2, although previous study reported that Glut2 was undetectable in rat ovary [17].

### Changes in Ovarian Weight

**Figure 2:**
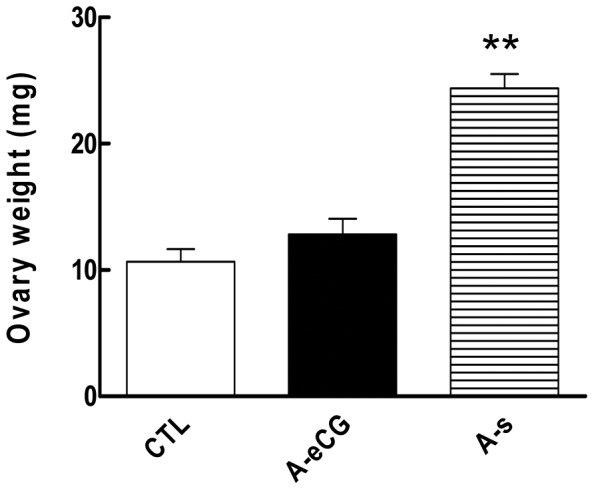
Effect of eCG antiserum injection on eCG-increased ovarian weight. Rats injected with 15 IU eCG and injected 24 h later with eCG antiserum (100 µl diluted in 0.9% saline, i.p., A-eCG) or preimmune serum (A-S). Ovaries were collected after anti-eCG antiserum treatment for 24 h. Results represent mean ± SEM of four experiments; * indicates significant difference from control (** P<0.01).

There was no difference between the ovarian weight of the anti-eCG antiserum rats and control rats. This suggests that there is no major alteration in ovarian density or shape. Moreover, the eCG antiserum caused inhibition of the significant increase in ovarian weight observed after eCG treatment [24.4±1.47 mg (eCG, A-s) vs. 12.8±1.94 mg (anti-eCG antiserum, A-eCG), P<0.01; Figure 2]. As follicle development concerned, large antral follicles at the preovulatory stage were shown in the eCG treated rat ovaries, were rarely present in ovaries from the anti-eCG antiserum-treated rats. Meanwhile, ovarian size in the antibody-treated group was notably smaller than that in the eCG treated group, which reflected that antibody treatment prevents the gain in ovarian weight.

### The regulation of eCG on Gluts expression

**Figure 3:**
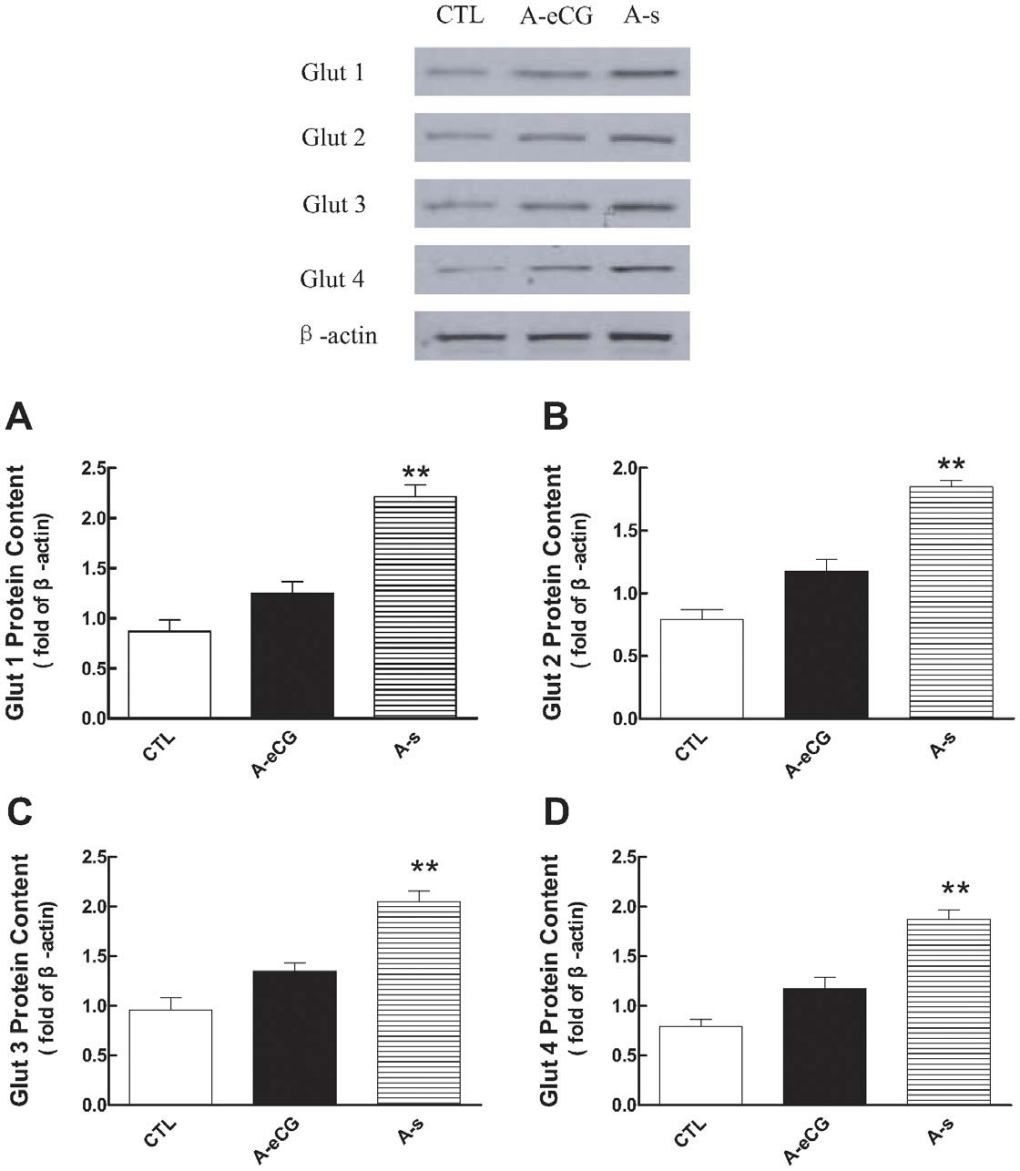
Western analysis of Glut1–4 protein content in ovary after eCG and or eCG antiserum injection. Ovarian protein extracts were prepared from control, eCG-primed rats treated with preimmune serum (A-S) or eCG antiserum (A-eCG), resolved by SDS-PAGE, and analyzed for Glut1–4 protein content with anti-Glut1–4 antibodies by Western blot. The Glut1–4 protein contents were normalized by β-actin. eCG significantly increased Glut 1–4 protein content compared with control group, which was abrogated by eCG-antiserum (Ab).

Since gonadotropin is very important factor which involved in follicular development, and facilitative glucose transporter (Glut) proteins mediate glucose uptake in ovary, and then we investigated whether eCG regulates Gluts expression in rat ovary. Western blot analysis revealed the presence of immunoreactive proteins corresponding to Glut1–4 in extracts of rat ovary. The protein content for Glut1 in eCG treatment group was higher than that of anti-eCG antiserum and control group [2.21±0.13 (eCG, A-s) vs 1.25±0.07 (anti-eCG antiserum, A-eCG), P<0.01, Figure 3A]. Consistent with its effects on the levels of other Glut isoforms, anti-eCG antiserum abrogated eCG-induced Glut2–4 protein content [Glut2: 1.79±0.09 (eCG, A-s) vs. 1.18±0.17 (anti-eCG antiserum, A-eCG); Glut3: 2.04±0.09 (eCG, A-s) vs. 1.35±0.05 (anti-eCG antiserum, A-eCG); Glut4: 1.87±0.09 (eCG, A-s) vs. 1.31±0.17 (anti-eCG antiserum, A-eCG); P<0.01, Figure 3B–3D].

**Figure 4:**
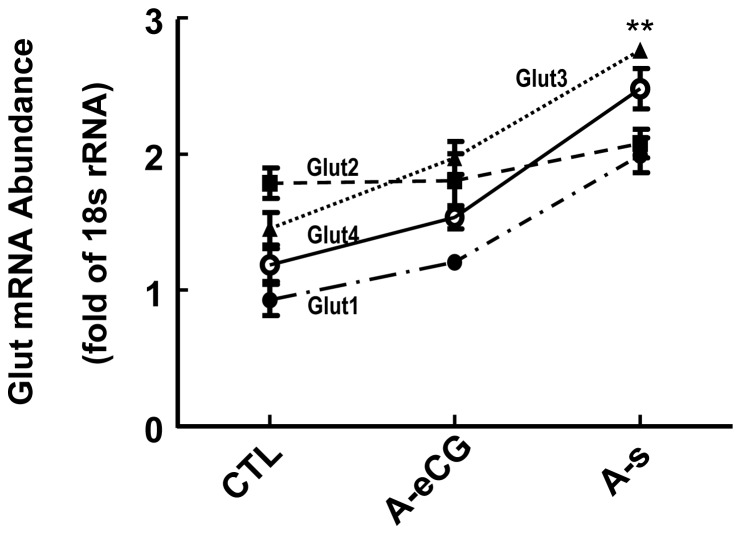
Effect of eCG on ovarian Glut1–4 mRNA content. The ovaries were also collected for Glut mRNA analysis by real-time PCR and RT-PCR. The Glut mRNA abundance were normalized by 18S rRNA and β-actin. Although eCG significantly increased Glut1, Glut3 and Glut4 mRNA level, these responses were abrogated by eCG antiserum. Neither eCG alone nor the combination of eCG and eCG antiserum elicited a significant response in the Glut2 mRNA regulation. **, P<0.01.

To better understand if the Glut1–4 proteins content are regulated at the mRNA level by hormone, Glut1–4 mRNA abundance were determined by qPCR. The results showed that gonadotropin significantly increased Glut1 mRNA level, which was abolished by anti-eCG antiserum [1.99±0.12 (A-s) vs. 1.21±0.06 (A-eCG), P<0.01, Figure 4]. Moreover, the same response were happened with the presence of eCG when compared to the respective anti-eCG antiserum (A-eCG) group in Glut3 [2.76±0.04 (A-s) vs. 1.97±0.11 (A-eCG), P<0.01, Figure 4] and Glut4 [2.48±0.14 (A-s) vs. 1.53±0.08 (A-eCG), P<0.01, Figure 4]. However, hormone had no effect on Glut2 mRNA content [2.07±0.07 (A-s) vs. 1.81±0.05 (A-eCG)], which is also confirmed by RT-PCR [Figure S1]. These results suggest that the regulation of ovarian Glut2 expression by hormone is at the translational level or via post-translational processing.

### The effect of eCG on blood glucose level

**Figure 5:**
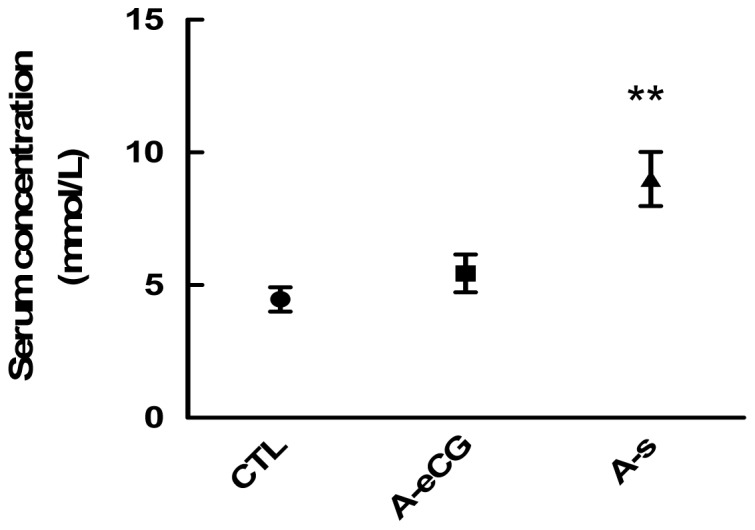
Biochemical analyses of serum glucose level among eCG, anti-eCG antiserum and control groups. Data are the mean ± SEM of four experiments. Rats were treated according to the methods. Blood were collected for biochemical analyses of serum glucose. Note that eCG significantly increased serum glucose level. However, anti-eCG antiserum abrogated eCG-induced serum glucose. **, P<0.01.

Since eCG regulated ovarian Gluts expression, we then examine whether it affects glucose level in blood. Rats blood were collected and glucose level in serum was examined among the groups. As indicated in Figure 5, serum glucose concentration were significantly increased in gonadotropin alone group compared with those in the control group [9.01±0.79 mmol/L (eCG, A-s) vs. 4.47±0.13 mmol/L (control), P<0.01]. The injection of anti-eCG antiserum reversed gonadotropin-induced high serum glucose level [9.01±0.79 mmol/L (eCG, A-s) vs. 5.44±0.49 mmol/L (anti-eCG antiserum, A-eCG), P<0.01]. Moreover, no significant differences were observed between anti-eCG antiserum and control groups.

### The effect of eCG on apoptosis in rat ovary

**Figure 6:**
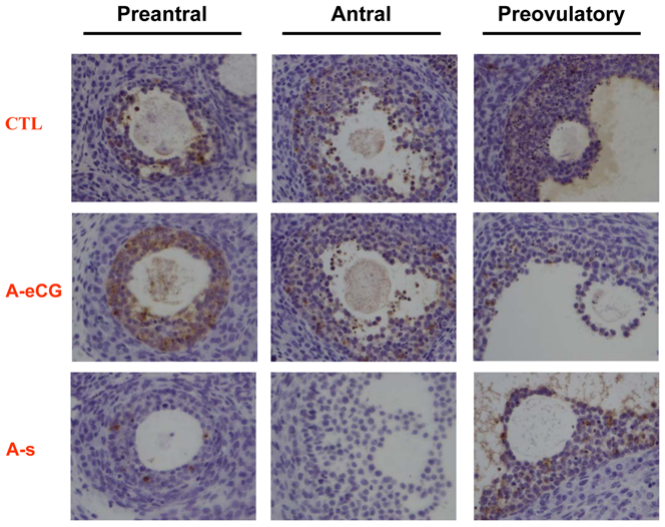
Effect of anti-eCG antiserum-induced apoptosis in granulosa cell. As described in the Material and Methods, the paraffin sections of the ovaries were prepared. In situ detection of apoptotic cells (TUNEL) on adjacent sections of preantral, antral and preovulatory follicle from control, eCG-primed rats treated with preimmune serum (A-S) or anti-eCG antiserum (A-eCG). Note that apoptotic cells can be seen scattered throughout the granulosa layer in different stage follicles in control group. Anti-eCG antiserum abolished eCG-induced protective effect from apoptosis. Higher magnification shows that the granulosa cell, but not theca cell and oocyte present TUNEL positive signal (Magnification, 400×). Each independent data came form five ovaries.

For further investigate the relationship between eCG and apoptosis in ovarian cell apoptosis during follicular development, adjacent histological sections of ovaries were examined for the cell death by TUNEL. Granulosa cell showed positive immunolabelling for TUNEL in different follicular development stage, and none of the TUNEL positive germ cell and theca cell were detected in any follicle type in the control ovarian sections. These are consistent with the previous report [22]. However, TUNEL-positive granulosa cells were undetectable in antral follicle in eCG alone treated group. In both preantral and prevoulatory follicle, TUNEL-positive cells were scattered throughout the granulosa layer. As shown in Figure 6, withdrawal of gonadotropin mediated by anti-eCG treatment in vivo resulted in a marked increase in granulosa cell apoptosis. Signal immunoreactivities in theca cell were also negligible.

## Discussion

In the present study, we found that Glut1 were widely expressed in rat ovary, which is consistent with previous study [6]. Meanwhile, the presence of Glut3 showed the same pattern with Glut1. Although Kol Shahar *et al.* reported that Glut2 and Glut4 were undetectable [17], our results showed that both of them were detected not only in granulosa cells but also in theca cells and oocyte for the first time. The difference maybe due to the different methods.

The localization, expression and regulation of the Glut1, Glut2, Glut3 and Glut4 are in tissue and cell-specific manner. Glut1 is ubiquitously expressed in all of organs, which is highly related to the basal level of glucose uptake in most cell types. In the rat ovary and uterus, the presence and regulation of Glut1 has also been reported [6], [25], [26]. Glut2 is a facilitative glucose transporter in the liver, pancreas, intestine, kidney, and brain. It has low affinity and high capacity, which ensure large bidirectional fluxes of glucose in and out the cell [27]. Glut3 is found in the brain, uterus, placenta, testis, prostate, ovary and some cancers [28]. Glut3 is important to tissues because of its higher affinity for glucose compared with Glut1, Glut2 and Glut4. Glut4 is probably the most studied protein among glucose transporter isoforms since it plays an important role in whole body glucose homeostasis and the pathogenisis of type II diabetes mellitus [9], [10], [11].

Glucose is an essential metabolic substrate, which is very important to maintain the normal ovary function and activity [6]. It is taken up into cells mediated by glucose transporter proteins [29]. Gluts are membrane proteins that expressed in tissue specific and regulated by metabolism and hormone. The physiological function of Gluts depends on their kinetic and substrate specificities [2]. For glucose, Glut1, Glut2 and Glut3 have high capacity with different Km, which are 1–2 mM, 10–20 mM and 0.3–1.4 mM respectively. GLUT4 has a Km of 1–7 mM and is closely tight with insulin [30]. These Km indicate that Glut3 has highest affinity for glucose than others, which ensures enough internal glucose concentration in the cell even if the peripheral glucose is lower. Certainly, Glut1, Glut2 and Glut4 simultaneously increase internal cell glucose uptake when glucose concentrations are relatively higher. These results indicated that glucose concentration in the ovary is ensured even if the Km of Gluts are different [30].

Ovary as a classical sex hormone responsive tissue, similar with breast, uterus and testis each have their unique functions and all respond in differing manners to GnRH (gonadotropin-releasing hormone), androgens, estrogen and progesterone. Thus it should no surprise that the Glut expression in each tissue is different.

GnRH is the main regulator of the hypothalamic-pituitary-gonadal axis, which stimulates the pituitary gonadotroph to synthesize and secrete FSH and LH. The latter exert different function in different follicular stage and ensure the normal ovarian cycle [31], [32]. The previous study showed that GnRH induced Glut1 mRNA expression levels and protein in the LβT2 cells, which is partly mediated by ERK activation [33]. Our study provides strong evidence that eCG increased not only Glut1 protein content but also mRNA level in rat ovary. Although gonadotropin failed to regulate Glut2 and Glut4 mRNA expression in the LβT2 gonadotroph cell line [33], which significantly increased the Glut2 and Glut4 protein level in rat ovary in present study. However, these up-regulation effects were reversed by anti-eCG antiserum. Although Glut2 mRNA abundance was not significantly changed by the hormonal treatment, the level of Glut2 protein was significantly increased, suggesting that the regulation of Glut2 protein may be at the translation level or via post-translational processing. Meanwhile, the increased Glut4 protein content is partially due to increased mRNA level.

In addition, our experiments showed that in comparison to the control and anti-eCG antiserum rats, the eCG alone treated rats had abundant Glut3 protein. The regulation of mRNA by hormone showed the same tendency with Glut3 protein content. Whether the increase in Glut mRNA abundance is due to increased gene transcription and stabilization of the message in response to gonadotropin, remains to be determined.

Base on these results, we are tempting to speculate that Glut isoforms may play meaningful role in ovarian follicle development in vivo. Moreover, Gluts expressed in different stage follicle indicated that Glut1–4 are engaged to transport enough glucose to ovarian cells, which is necessary to follicle development, terminal follicular maturation, and the resumption of meiosis.

Glucose is transported from blood to the tumor cells, which must be delivered across gluts on the vascular endothelium and then on the plasma membrane of the tumor cells [34]. Many studies have shown a tighter association between Glut presence and different cancer, which means Glut plays an important role in transporting glucose to tissues. In addition, GnRH stimulates glucose uptake in the gonadotroph [33]. Previous studies have demonstrated that glucose regulates Glut1 expression in tissue culture [35]. Glut may play a vital part in supplying energy to the developing follicle and oocyte by facilitating glucose uptake. In this study, we also examined serum glucose level. Consistent with the high expression of Glut1–4, eCG had a strong stimulatory effect on serum glucose increases. However, this effect was reversed by anti-eCG antiserum. eCG-induced higher serum glucose level is possibly due to its stimulated glucagon release [36]. Moreover, high serum glucose level maybe relatively regulates Glut isoform expression, which enhances glucose uptake in ovarian cells.

Our results also showed that eCG significantly induced ovarian weight. These results indicate that gonadotropin affects glucose uptake and induces ovarian development by increasing Glut gene expression, and or through affecting Glut localization/translocation. Moreover, gonadotropin stimulated ovary development partially attribute to its own receptor expressed in ovary [37], [38]. The further study may focus on the detailed analyses of glucose transporters in ovary to better elucidate the mechanisms by which these genes and proteins are regulated in normal and pathogenic situation. The eCG antiserum caused a inhibition of the significant increase in ovarian weight after eCG treatment. These results indicated that eCG antiserum inhibits Gluts expression in rat ovary, which induced ovarian weight decrease.

Apoptosis, a physiological form of cell death, is the cellular mechanism of ovarian follicular atresia. In the present study, TUNEL was used to detected apoptotic cell in rat ovary. In preantral, antral and preovulatory follicles, TUNEL positive staining was present in the granulosa cells, which is consistent with previous report [22]. The present results also indicate gonadotropin inhibited the apoptotic signal in granulosa cells. Interestingly, TUNEL-positive cells were scattered throughout the granulosa layer in preantral follicle. The latter is in the gonadotropin-independent phase maybe the explanation of the results. However, the anti-eCG antiserum abrogated the eCG protective effect from apoptosis. Apoptosis in granulosa cells following anti-eCG antiserum-induced gonadotropin withdrawal may be due to Fas and FasL expression in granulosa cells [22]. However, no apoptosis was observed in theca cells. This gonadotropin initiates the differentiation of the granulosa cells and renders them especially susceptible to apoptosis. In additional, theca cells may be deficient in some component of the cascade of signaling molecules activated by ligand binding to Fas [22].

In conclusion, our present study found that Glut1–4 expressed in rat ovary. As such, this report establishes for the first time gonadotropin up-regulates Gluts content in rat ovary. Consistent with the high expression of Glut1–4, gonadotropin had strong stimulatory effect on serum glucose increase. Meanwhile, gonadotropin induces follicle development partially through inhibiting granulosa cells apoptosis. Gluts are also been taken as markers of follicular development. Further study will focus on the mechanism how gonadotropin regulates Gluts expression and glucose uptake in ovarian cells.

## Materials and Methods

### Materials

Culture media were purchased from Gibco Bethesda Research Laboratories (Grand Island, NY, USA). eCG (equine chorionic gonadotropin) and Tween 20 were obtained from Sigma Chemical Co. (St. Louis, MO). The 6-well plate was product of Becton Dickinson (Becton Dickinson Labware, USA). The enhanced chemiluminescence (ECL) detection kit was obtained from Amersham Life Science (Oakville, ON, Canada). Acrylamide (electrophoresis grade), N,N′-methylene-bis- acrylamide, ammonium persulfate, glycine, SDS-PAGE prestained molecular weight standards (low range), nitrocellulose membranes, and horse radish peroxidase (HRP)-conjugated anti-rabbit and anti-mouse immunoglobulin (Ig) G were products of Bio-Rad (Richmond, USA). Rhodamine-conjugated goat anti-rabbit IgG, and mouse IgG were product of Santa Cruz Biotechnology, Inc. (Santa Cruz, USA). Preimmune serum (normal rabbit serum) and the anti-eCG antibody were from Zhongshan Inc. (Zhongshan Company, Beijing, China)

Rabbit polyclonal anti-rat Glut1 (ab53767), rabbit polyclonal anti-rat Glut3 (ab41525) and rabbit polyclonal anti-rat Glut4 (ab65976) were purchased from Abcam (Abcam, USA). Goat polyclonal anti-rat Glut2 (sc-7580) were from Santa Cruz Biotechnology, Inc. (Santa Cruz, USA). These antibodies were used for IHC.

Goat polyclonal anti-rat Glut1 (sc-1605), rabbit polyclonal anti-rat Glut2 (sc-9117) and goat polyclonal anti-rat Glut3 (sc-31840) and mouse monoclonal anti-rat β-actin (sc-81178) were also purchased from Santa Cruz Biotechnology, Inc. (Santa Cruz, USA). Mouse monoclonal anti-rat Glut4 was from Cell Signaling (Cell Signaling, USA). The above antibodies were used to detect protein level by Western Blot analysis.

Random decamer primers were from Ambion, Inc. (Austin, TX). Ribonuclease (RNase) inhibitor, RevertAid H Minus M-MULV RT Enzyme, 5× reaction buffer, and dNTP were from Fermentas International Inc. (Burlington, Ontario, Canada). RNeasy Micro kit, deoxyribonuclease I in RNase-free deoxyribonuclease set and QuantiTect SYBR Green PCR kit were purchased from QIAGEN Inc. (Mississauga, Beijing, China). PCR primers for Glut1, Glut2, Glut3, Glut4 and 18S rRNA were from Sunbiotech Inc. (Beijing Sunbiotech Co., Ltd. China).

### Animal Treatments

Sprague-Dawley rats were purchased from the Beijing Vital Laboratory Animal Technology Co. (Beijing, China). All procedures were carried out in accordance with the Guidelines for the Care and Use of Laboratory Animals and China Council on Animal Care and were approved by the Institutional Animal Care and Use Committee of Capital Normal University. All animals were caged in a controlled environment with a 12/12-h light/dark cycle and received pathogen-free water and food for maintenance. Twenty-two to twenty-five day old immature female Sprague-Dawley rats were injected subcutaneously with 15 international units (IU) of eCG in 100 µl of phosphate buffered saline (PBS) containing 0.2% (w/v) bovine serum albumin (BSA) or PBS containing 0.2% (w/v) bovine serum albumin (BSA) (control). 24 h later, the control rats were injected preimmune serum (100 µl diluted 1∶10 in 0.9% saline, i.p.). eCG-injected group were divided into two groups randomly and injected anti-eCG antiserum (100 µl diluted 1∶10 in 0.9% saline, i.p., group defined as A-eCG) and preimmune serum (100 µl, diluted 1∶10 in 0.9% saline, i.p., group defined as A-s) respectively. This protocol has been used to neutralize gonadotropin effect and induce atresia in previous study [21], [22]. After 24 h, animals were killed by cervical dislocation. Each group includes at least 10 rats.

### Immunochemistry

As described in the previous section, rats were killed and ovaries quickly removed, cleared of adhering fat. Ovaries were isolated from different groups and fixed in 10% neutral buffered formalin were dehydrated through a graded series of ethanol (70–100%), cleared in xylene, embedded in paraffin, and sectioned (4–5 µm). The sections were deparaffinized in xylene and then rehydrated through a graded series of ethanol (100–50%) to distilled water. After dewaxing, rehydration, and antigen retrieval with 0.01% sodium citrate buffer (pH 6.0), ovaries were immunostained with rabbit anti-Glut or goat anti-Glut antibody (1∶500). Subsequently, these sections were stained with biotinylated rabbit anti-goat or goat anti- rabbit IgG (pv-6003; Zhongshan Company, Beijing, China) and then incubated with ABC complex (Zhongshan) for 1 min. Finally, the sections were counterstained with hematoxylin. Non-immune goat serum was used as the controls.

### Protein Extraction and Western Blot Analysis

Western blot analysis was performed as described previously [23]. Ovaries were collected and homogenized in buffer containing tris (hydroxymethyl) aminomethane hydrochloride (Tris-HCl 20 mM, pH 7.5), sucrose (0.25 M), MgCl_2_ (2.5 mM), EDTA (2.5 mM), KCl (10 mM), thimerosal (0.02%), and a protease inhibitor cocktail (Sigma-Aldrich, MO). The homogenate was centrifuged for 10 min at 800 g; the supernatant was collected and centrifuged again (14,000× g, 4°C, 30 min).

All protein extraction was carried out on ice, and the protein content of the supernatant (cell lysate) was determined with the Bio-Rad DC protein assay kit. Aliquots of proteins (15 µg) were resolved by SDS-PAGE (10%) and electrotransferred to nitrocellulose membranes. The membranes were then blocked (RT, 1 h) with blotto [Tris-buffered saline (pH 8.0) with 0.05% Tween 20 (TBS-T), 5% dehydrated nonfat milk powder]; they were then incubated (4°C, overnight) with blotto containing 0.1 g/ml Glut1 (1∶1000), Glut2 (1∶1000), Glut3 (1∶1000), Glut4 (1∶1000), β-actin (1∶10,000) antibody washed in TBS-T (3×5 min), incubated in HRP-conjugated secondary antibody (1∶3000, 1∶3000, 1∶1000, 1∶1000, 1∶1000, respectively) in blotto, and washed again in TBS-T. Peroxidase activity was visualized with the ECL kit according to the manufacturer's instructions, and protein content was determined by densitometrically scanning the exposed x-ray film.

### RNA extraction, cDNA synthesis, and real-time PCR analysis

Total RNAs were extracted from ovary that were collected from the above indicated groups using RNeasy Micro kit. Briefly, 0.2 µg total RNAs were reverse transcribed in a final volume of 20 µl solution containing 4 µl 5×reaction buffer, 2 µl 10 mM dNTP, 20 U of RNase inhibitor, 200 U RevertAid H Minus M-MULV RT enzyme, random decamer primers, and RNase free H_2_O. Quantitative PCR analysis for Glut1, Glut2, Glut3, Glut4 and 18S rRNA were performed, using a LightCycler 2.0 System (Roche Diagnostics). The Glut1 primers used for amplification were a 5′ forward primer (5′-TGGCCAAGGACACACGAATACTGA-3′) and a 3′ reverse primer (5′-TGGAAGAGACAGGAATGGGCGAAT-3′). The Glut2 primer sequences were 5′-TAGTCAGATTGCTGGCCTCAGCTT-3′ (5′ forward primer) and 5′- TTGCCCTGACTTCCTCTTCCAACT-3′ (3′ reverse primer). The Glut3 primers used for amplification were a 5′ forward primer (5′- TGGCTACAACACCGGAGTCATCAA-3′) and a 3′ reverse primer (5′-CTGCCAAAGCGGTTGACAAAGAGT-3′). The Glut4 primers used for amplification were a 5′ forward primer (5′ -AGTTGGAAAGAGAGCGTCCACTGT-3′) and a 3′ reverse primer (5′- GCTGCAGCACCACTGCAATAATCA-3′). The 18S rRNA primer sequences used were a 5′ forward primer (5′-CGCGGTTCTATTTTGTTGGT-3′) and a 3′ reverse primer (5′-AGTCGGCATCGTTTATGGTC-3′). Amplification reaction was then performed using the QuantiTect SYBR Green PCR kit. The thermal cycling conditions were comprised of an initial denaturation step (95°C, 15 min) followed by 42 cycles amplification for Glut1–4 and 40 cycles amplification for 18S rRNA (95°C for 15 sec, 58°C for 20 sec, and 72°C for 30 sec, respectively). PCR products were subsequently melted at 60°C for 30 sec. The melting curve analysis shows a single peak with no primer-dimers at the described PCR working conditions for Glut1–4 and 18S primer sets, respectively. A standard curve was included for each gene. All the real time-PCR assay for Glut1–4 mRNA abundance were quantified within the linear range, normalized against their respective 18S rRNA mRNA [24]. PCR without reverse-transcribed cDNA were used as negative controls.

### RT-PCR (Reverse transcriptase polymerase chain reaction analysis)

For RT-PCP analysis, the Glut2 sense and antisense PCR primers were 5′- ACCTCCAGGATGAAGGAAACAGCA-3′ and 5′-TTCAGTGAAAGCGCCAGTTTGTGG-3′, respectively. Product size is 328 bp. The β-actin sense and antisense primers were 5′-CCA AGGCCAACCGCGAGAAGATGAC-3′ and 5′-AGGGTACATGGTGGTGCCGCCAGAC-3′, respectively. Product size is 578 bp. Amplification was conducted in a total of 25 µl. After an initial denaturation step at 94°C for 10 min, temperature cycling was initiated as follows: 94°C for 40 sec, 58°C for 30 sec, and 72°C for 30 sec during 30 cycles for Glut2 and β-actin. An extra extension at 72°C was performed for 10 min. Samples were run on a 1.5% agarose gel and visualized by staining with ethidium bromide. Densitometry was carried out using a Bio-Rad GelDoc image acquisition system and Quantity One (v4.3) quantitation software (Bio-Rad, Hercules, CA, United States).

### Blood analyses

Blood was collected and kept at 37°C for 1 h, and then centrifuged at 200 g for 15 min. The serum was separated and transferred to a sterilized tube. The serum was either used immediately for blood biochemical analysis. Glucose was assayed by Technicon RA-1000 (Bayer, Tarrytown, NY, USA) using Zhongsheng kits (Zhongsheng Biotechnology, Beijing, China). The intra- and inter-assay coefficients of variation were 10%.

### TUNEL

To identify cell death, TUNEL (TdT-mediated dUTP nick end-labeling) was applied in the paraffin sections of the ovaries. Briefly, each slide was incubated with proteinase K (10 µg/ml in 20 mM Tris and 2 mM CaCl2, pH 7.4; 37°C, 30 min), and endogenous peroxidase activity was removed by treatment with 0.3% H2O2 (RT, 20 min). After rinsing in distilled water for 15 min, the sections were soaked in TdT buffer (25 mM Tris-HCl, pH 6.6, 200 mM sodium cacodylate, 5 mM cobalt chloride, 250 µg/ml BSA) for 15 min and then incubated in 50 ml of TdT buffer containing 10UTdT and 1 nmol biotinylated 16-dUTP in a humidified chamber (37°C, 60 min). The biotinylated dUTP molecules incorporated into nuclear DNA were reacted with HRP conjugated streptavidin (1∶100; RT, 30 min). After further washing in PBS (15 min), the peroxidase coloring reaction was performed by immersing sections (10 min) in 0.05 M Tris-HCl buffer (pH 7.6) containing 0.3 mg/ml DAB (diaminobenzidine tetrahydrochloride), 10 mM imidazole, and 0.003% H2O2. Sections were not counterstained and were dehydrated through a graded series of ethanol (50–100%) to xylene and mounted for examination.

### Statistical Analysis

Results are presented as means ± SEM of at least three independent experiments, as detailed in the figure legends. All data were subjected to one way (repeated-measure) ANOVA (Prism 5.0 statistical software; GraphPad Software, Inc., San Diego, CA). Significant differences between treatment groups were determined by the Tukey's test. Statistical significance was inferred at P<0.05.

## Supporting Information

Figure S1 Effect of eCG on ovarian Glut2 mRNA content corresponding to the Figure 4.
(TIF)
